# Prognostic Factors and a Nomogram Predicting Overall Survival and Cancer-Specific Survival for Patients with Collecting Duct Renal Cell Carcinoma

**DOI:** 10.1155/2021/6736008

**Published:** 2021-11-11

**Authors:** Ruotao Xiao, Cheng Liu, Wei He, Lulin Ma

**Affiliations:** Department of Urology, Peking University Third Hospital, Beijing, China

## Abstract

**Background:**

Collecting duct renal cell carcinoma (CDRCC) is a rare type of renal cancer characterized by a poor prognosis. The aim of this work was to develop a nomogram predicting the overall survival (OS) and cancer-specific survival (CSS) for patients with CDRCC.

**Methods:**

A total of 324 eligible patients diagnosed with CDRCC from 2004 to 2015 were identified using the data from the Surveillance, Epidemiology, and End Results (SEER) database. The Kaplan-Meier curve was used to estimate the 1-, 3-, and 5-year OS and CSS of these patients. Univariate and multivariate Cox regression models were performed to identify the independent risk factors associated with OS and CSS. The nomogram was developed based on these factors and evaluated by the concordance index (C-index) and calibration curves using the bootstrap resample method. The predictive accuracy of the nomogram was also compared with the manual of the American Joint Committee on Cancer (AJCC).

**Results:**

The estimated 1-, -3, and 5-year OS and CSS rates in the analytic cohorts were 56.4% and 60%, 32.5% and 37.3%, and 28.7% and 33.6%, respectively. The multivariate model revealed that age, tumor size, tumor grade, N stage, M stage, surgical type, and chemotherapy were independent predicted factors for OS, while tumor size, tumor grade, N stage, M stage, surgical type, and chemotherapy were independently linked to CSS. A nomogram was developed using these factors with relatively good discrimination and calibration. The C-index for OS and CSS was 0.764 (95% CI: 0.735~0.793) and 0.783 (95% CI: 0.754~0.812), which was superior to the AJCC stage (C-index: 0.685 (95% CI: 0.654~0.716) and 0.703 (95% CI: 0.672~0.734)). Patients were divided into low-risk, intermediate-risk, and high-risk groups according to the total points calculated by the nomogram. Patients in the low-risk group (97 mo and not reached) experienced significantly long median OS and CSS compared to the intermediate-risk (17 mo and 18 mo) and high-risk groups (5 mo for both). The calibration curves showed a good agreement between the predicted and actual probability related to OS and CSS.

**Conclusion:**

CDRCC has an aggressively biologic behavior with relatively poor prognosis. A survival prediction nomogram making an individualized evaluation of OS and CSS in patients with CDRCC was presented, potentially helping urologists to make a better risk stratification.

## 1. Introduction

Renal cell carcinoma (RCC) is one of the most common human malignancies worldwide, and its incidence steadily increased in most countries [1]. The prognosis in patients with RCC is generally favorable, with 5-year overall survival (OS) and cancer-specific (CSS) rates of 73.2%~87.9% and 84%~95%, respectively [2, 3]. Nowadays, many prognostic models like UISS, SSIGN, and Leibovich have been established to predict the oncologic outcome in patients with RCC [4–6]. The cancer stage manual American Joint Committee on Cancer (AJCC) is the most commonly used model in clinical practice and includes the T stage, N stage, and M stage and comprehensively divided patients into I~IV groups [7]. However, the above models were established mainly based on a population of a clear cell subtype. It is currently unknown whether it is still accurate enough to predict the prognosis for other subtypes of RCC.

Collecting duct renal cell carcinoma (CDRCC) is a rare but aggressive histologic subtype of RCC, estimated to comprise less than 1% of the entire cohort [8–11]. Few studies explored the survival outcomes and prognostic factors of CDRCC due to the rarity of this subtype. Most studies regarding CDRCC are based on case reports and series from a single center with a limited sample size, which cannot provide comprehensive insights for urologist [12–14]. The largest series to date include 577 CDRCC collected from the National Cancer Database, which revealed that this subtype is drastically more aggressive than clear cell carcinoma (CCRCC) [9]. Patients with CDRCC have higher tumor grade (G3+G4: 62.3% *vs.* 24.4%), advanced T stage (T3+T4: 57% *vs.* 20.9%), N stage (N1: 27.7% *vs.* 2.3%), and M stage (M1: 32.1% *vs.* 13%) and shorter median survival (12.3 mo *vs.* 122.5 mo) as compared to those with CCRCC [9]. A recent study performed a retrospective analysis on 69785 patients with RCC including 280 patients with CDRCC and revealed that CDRCC not only has more advanced TNM stage than CCRCC but also shows higher cancer-specific mortality even after matching with G4 CCRCC (HR: 1.6, *P* < 0.01) [15]. Besides, CDRCC prognosis varies widely among previous studies, with reported median survival ranging from 13 months to 4.9 years [8–11, 15, 16]. The main reason for the variety of the prognosis could be the heterogenous risks of patients among studies. Therefore, it is an urgent matter for a urologist to establish a specific prognostic model to assess risk stratification in patients with CDRCC to accurately inform patients on their long-term survival. The rarity of this subtype makes the study difficult in a large-scale prospective manner. Therefore, in this work, a nomogram was developed to predict OS and CSS in patients with CDRCC using the Surveillance, Epidemiology, and End Results (SEER) database.

## 2. Materials and Methods

### 2.1. Study Population

The SEER database is a population-based cancer database that collects data from 18 registries among 14 states and covers around 28% of the population across the USA. A retrospective cohort study was conducted using the SEER database of the National Cancer Institute (http://seer.cancer.gov/). The datasets of patients in the present research were downloaded from the SEER∗Stat 8.3.9 software. Patients diagnosed with CDRCC (histological diagnostic code 8319/03 in the International Classification of Diseases for Oncology, 3rd Edition (ICD-O-3)) from 2004 to 2015 were included in this study. Patients with missing data on baseline characteristics and follow-up were excluded. Finally, 324 eligible patients were included for further analysis.

### 2.2. Variable Collection

The considered variables were age at diagnosis, race, gender, tumor laterality, year of diagnosis, marital status, tumor grade, tumor size, AJCC stage, clinical T stage, N stage, M stage, surgical type, radiotherapy, chemotherapy, follow-up time, cancer-specific death, and death of any other cause. Age at diagnosis, tumor size, and follow-up time were recorded as continuous variables. The others were recorded as categorical variables. Patients were restaged according to the 8th edition AJCC Cancer Staging Manual. The surgical type was recorded as “without,” “nephron-sparing surgery (NSS),” and “radical nephrectomy (RN).” The adjuvant treatment including chemotherapy and radiotherapy was recorded as “with or without.” The primary outcome of this study was the overall survival (OS), which was defined as the time interval between the day of diagnosis and death of any other cause. The secondary outcome in this study was cancer-specific survival (CSS), which was defined as the time interval between the day of diagnosis and cancer-specific death.

### 2.3. Statistical Analysis

Nomogram establishment and calibration were performed by using R software (Version 4.0.3) using the “rms” package, and other statistical analyses were performed using SPSS (version 26, IBM, Armonk, NY). The continuous variable was reported as median with interquartile ranges (IQR), and the categorical variable was reported as the whole numbers and proportions. The Kaplan-Meier method was used to estimate the 1-year, 3-year, and 5-year OS and CSS in the study cohort. Univariable and multivariable Cox proportional hazard regressions were performed to identify the independent prognostic factors associated with OS and CSS (forward stepwise selection methods). The selected independent factors were incorporated in the nomograms to predict the probability of 1-year, 3-year, and 5-year OS and CSS. The discrimination of the nomogram was measured by the concordance index (C-index), which ranges from 0.5 (no predictive power) to 1 (perfect prediction) [17]. The Kaplan-Meier curve and log-rank test were also performed to evaluate the ability of the risk stratification of the nomogram associated with OS and CSS. The calibration was evaluated using a calibration curve, which was assessed between the observed outcome probability and the nomogram-predicted probability, with a bootstrap resample of 1000 times. Besides, the C-index of the conventional AJCC stage was also calculated and compared to that of the established nomogram. All tests were two-sided, and *P* < 0.05 was considered statistically significant.

## 3. Results

### 3.1. Patients' Characteristics

A total of 324 patients were identified and included in this study. The median age of CDRCC was 61.5 (IQR: 53~72) years, and 223 (68.8%) cases were male (Table 1). The majority of the patients had high tumor grade (G3+G4: 63.3%), advanced T stage (T3+T4: 63.3%), and AJCC stage (III~IV: 69.2%). Besides, 116 (35.8%) and 115 (35.5%) patients had lymph node metastasis and distant metastasis, respectively. Among these patients, 280 (86.4%) underwent surgery, and among them, 20 (5.1%) and 260 (81.3%) received NSS and RN, respectively. Besides, 36 (11.1%) cases were subjected to radiotherapy and 88 (27.2%) cases were treated with chemotherapy.

The patient follow-up was of a median time of 17 (IQR: 6~55.8) months. Finally, 249 (76.9%) patients reported death with an estimated 1-year, 3-year, and 5-year OS rate of 56.4%, 32.5%, and 28.7%, respectively. Besides, 208 (64.2%) patients reported cancer-specific death with an estimated 1-year, 3-year, and 5-year CSS rate of 60%, 37.3%, and 33.6%, respectively.

### 3.2. Univariate and Multivariate Prognostic Analyses

The univariate and multivariate COX regression models were performed to analyze factors in predicting OS and CSS to identify the prognostic factors in patients with CDRCC (Tables 2 and 3). The univariate analysis revealed that factors including age, tumor size, tumor grade, T stage, N stage, M stage, surgical type, radiotherapy, and chemotherapy were associated with OS, while marital status, tumor size, tumor grade, T stage, N stage, M stage, surgical type, radiotherapy, and chemotherapy were associated with CSS. The multivariate analysis revealed that age, tumor size, tumor grade, N stage, M stage, surgery, and chemotherapy were independent predicted OS, while tumor size, tumor grade, N stage, M stage, surgery, and chemotherapy were independently linked to CSS.

### 3.3. Nomogram Development and Validation

**Figure 1:**
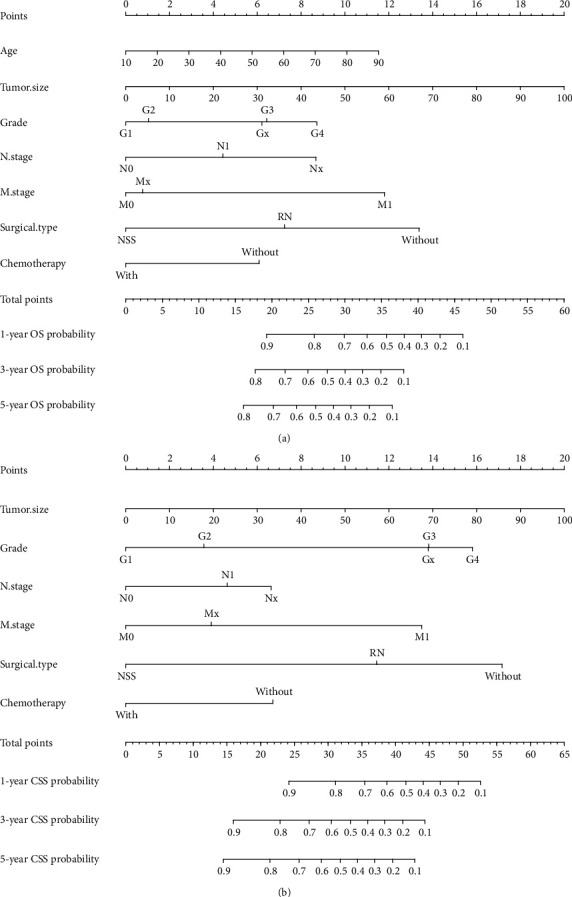
The nomogram to predict overall survival (a) and cancer-specific survival (b) on seven independent prognostic factors.

**Figure 2:**
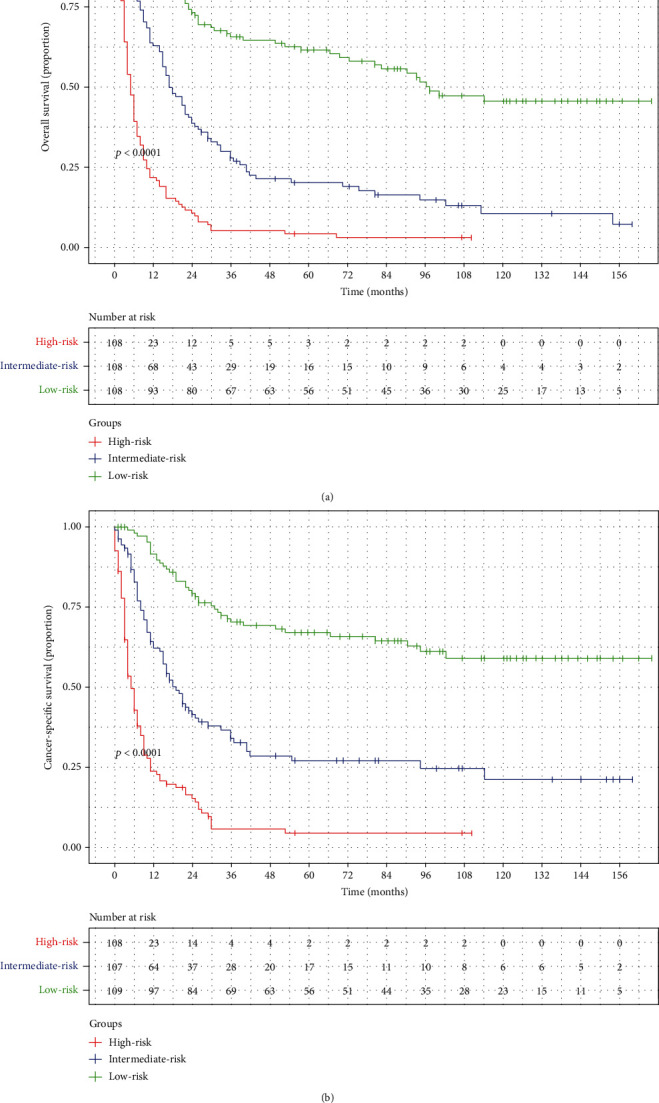
Kaplan-Meier survival curves of overall survival (a) and cancer-specific survival (b) stratified by low-risk, intermediate-risk, and high-risk patients according to nomograms.

**Figure 3:**
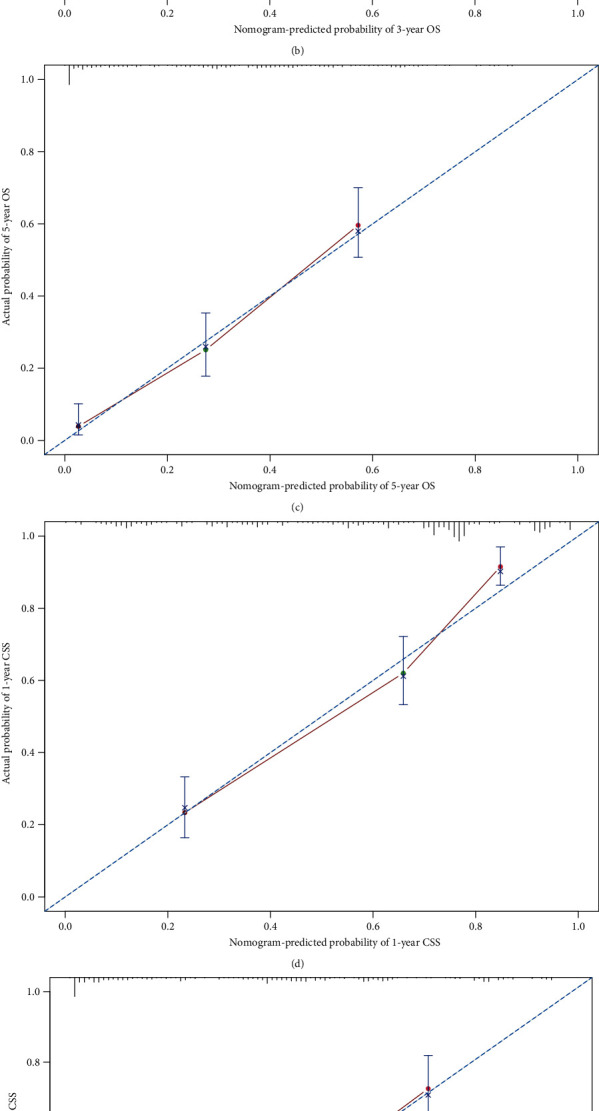
Calibration curves showing the probability of 1-, 3-, and 5-year overall survival (a–c) and cancer-specific survival (d–f) between the nomogram prediction and the actual observation.

Two nomograms incorporating the above-mentioned independent prognostic factors were developed to predict the 1-, 3-, and 5-year OS and CSS in patients with CDRCC (Figure 1). The C-index of the nomogram predicting OS and CSS was 0.764 (95% CI: 0.735~0.793) and 0.783 (95% CI: 0.754~0.812), respectively. The predicted probability of OS and CSS was then plotted as Kaplan-Meier curves stratified by the tertile of the predicted probability calculated from the nomogram to further assess the discriminative ability of the nomogram. The median OS and CSS were significantly longer in the low-risk (first tertile: 97 mo and not reached) group than in the intermediate-risk (second tertile: 17 mo and 18 mo) and high-risk (third tertile: 5 mo for both) groups, which also indicated a good discrimination of the established nomograms (Figures 2(a) and 2(b)). The accuracy of the nomogram and potential model overfit were assessed by the bootstrap validation with 1000 resamplings. The calibration curves showed a good agreement between the predicted and actual probability related to OS and CSS (Figure 3).

**Figure 4:**
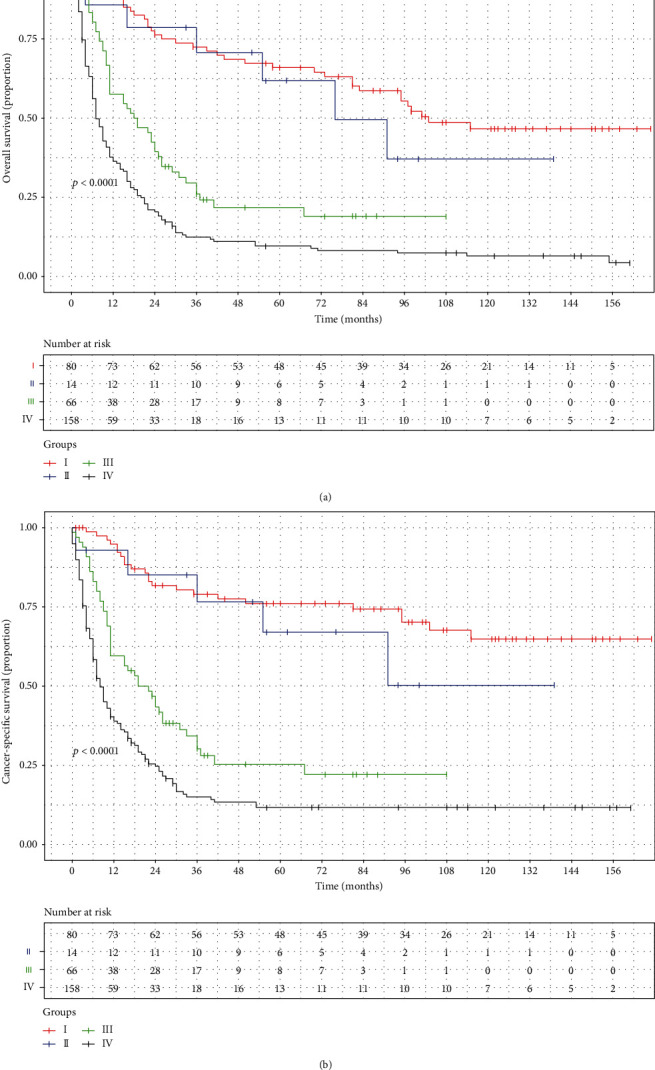
Kaplan-Meier survival curves of overall survival (a) and cancer-specific survival (b) stratified by the American Joint Committee on Cancer stage.

Our nomogram was compared with the conventional AJCC stage to further verify the predictive accuracy. The C-index of the conventional AJCC stage was 0.685 (95% CI: 0.654~0.716) and 0.703 (95% CI: 0.672~0.734) in predicting OS and CSS, which was significantly inferior to that of our nomogram. Besides, the Kaplan-Meier curves demonstrated that the AJCC stage could stratify patients between stages I~II and stages III~IV, whereas it was unsatisfactory in stratifying patients among stages I~II (Figures 4(a) and 4(b)). Therefore, the established nomogram had better risk stratification than the conventional AJCC stage.

## 4. Discussion

Not only is CDRCC a rare disease but also it has an aggressive biological behavior compared to the conventional RCC. The survival of CDRCC patients can widely vary, reflecting the prognostic heterogeneity associated with this disease [8–11, 15, 16]. An accurate prognostic model is critically important to inform patients about their long-term risk and guide the follow-up schedule. In the present study, two nomograms were proposed using the SEER database that can numerically predict the individual OS and CSS in patients with CDRCC based on clinicopathologic parameters and treatment modality. Patients could be divided into three risk groups according to the nomogram, with completely different survival prognoses. In the high-risk group, the median OS and CSS were only 5 mo, drastically shorter than those in the intermediate- and low-risk groups. Besides, our nomograms showed their superiority than the conventional AJCC stage system. Thus, the established nomograms could help urologists in performing a better risk stratification in patients with CDRCC.

Several larger series to date demonstrated the clinicopathological characteristics and prognosis of CDRCC patients [8–11, 15, 16]. Tokuda et al. [11] retrospectively analyze 81 cases from a multicenter in Japan and found that the lymph node and distant metastasis rate in these patients were 44.2% and 32.1%, respectively. Besides, the 1-, 3-, and 5-year CSS rates were only 69%, 45.3%, and 34.3%, respectively [11]. Karakiewicz et al. [16] analyzed 41 CDRCC and 5246 CCRCC, and their results revealed that CDRCC patients more often had higher tumor grade (G3+G4: 78% *vs.* 30%), advanced N (N1~2: 49% *vs.* 8%), and M stage (M1: 19% *vs* 14%), but cancer-specific mortality was not different between CDRCC and CCRCC after comparing the baseline data. In contrast, Wright et al. [8] and Sui et al. [9] demonstrated that CDRCC patients had not only advanced T, N, and M stages at the time of diagnosis but also adverse prognosis in comparison with CCRCC patients. Although the present study failed to compare the survival difference between CDRCC and CCRCC, the proportion of advanced T, N, and M stages and higher tumor grade in our study were higher or at least similar to those in the above results. Besides, it is worth noting than the 5-year OS and CSS rates in our study cohort were only 28.7% and 33.6%, respectively, which were drastically lower than the survival rates of CCRCC (73.2% and 84%) reported in previous studies [2, 3].

The treatment modality of this rare disease is relatively difficult due to its aggressively biologic behavior. Surgery remains the mainstay option for most urologists, especially for patients who are in localized stages. The largest series suggested that those who underwent surgery have more survival benefits compared to those who did not (HR: 0.13, *P* = 0.005), and our findings are also consistent with those of [10]. The subgroup analysis revealed that those who were diagnosed with metastatic CDRCC and treated with cytoreductive surgery could also experience longer survival compared to those who did not (median survival: 4.4 mo *vs.* 1.5 mo), which is in agreement with another study [10, 18]. Besides, many studies suggested that patients with CDRCC also benefit from chemotherapy [19–21]. In our study, chemotherapy was an independent protective factor associated with both OS and CSS. To our knowledge, conventional CCRCC is resistant to chemotherapy. However, CDRCC is derived from the distal nephron, sharing many similarities with urothelial tract carcinoma [22]. The gemcitabine+platinum (GC) regimen, which is the classic chemotherapeutic regimen for urothelial tract carcinoma, has been used in clinical practice as first-line adjuvant chemotherapy in patients with metastatic CDRCC. In a prospective phase II study of the GC regimen in 23 cases of metastatic CDRCC, the object response rate was 26% (1 complete and 5 partial responses) [19]. Another study analyzed 5 metastatic CDRCC receiving the bevacizumab+GC regimen and found a partial response in 3 cases, stable response in 1 case, and complete remission in 1 case [20]. A recent clinical trial enrolled 26 patients with metastatic CDRCC treated with the sorafenib+GC regimen and found that the object response rate was 30.8% [21]. In general, the GC-based chemotherapy regimen is not encouraging in the above studies. Several case reports suggested that metastatic CDRCC can potentially benefit from several treatments including cabozantinib [23], nivolumab [24], nivolumab+ipilimumab [25], personalized neoantigen-based immunotherapy [26], and HER2 blockade [27]. However, the evidence level of these therapies was relatively low. Pagani et al. [28] reviewed the literature and summarized the current treatment options and ongoing phase II clinical trials focusing on CDRCC. A phase II trial conducted in France enrolled 41 patients with metastatic CDRCC treated with the bevacizumab+GC regimen [29]. The trial was completed, but the results have not been reported. Another phase II trial conducted in Italy evaluating the activity and safety of cabozantinib as first-line treatment for metastatic CDRCC patients was also completed, and researchers were waiting for their results [30]. However, up to now, the management options of CDRCC continue to be investigated and evolve, since the optimal treatment remains unclear.

Several prognostic models had been established to predict the prognosis in patients with RCC [4–6]. However, these models were largely focused on CCRCC, neglecting the significant subset of patients with nonclear cell histology. In 2018, Leibovich et al. [3] established histology-specific prognostic models focusing on three major histologic subtypes (CCRCC, papillary RCC, and chromophobe RCC), but CDRCC was not included due to its rarity. As mentioned above, the clinicopathological characteristics and treatment modality of CDRCC are largely different from those of CCRCC. Thus, the prognostic model of CDRCC should also be unique. May et al. [10] were the first who developed a prognostic model based on the American Society of Anesthesiologists (ASA) score 3–4, tumor size greater than 7 cm, stage M1, Fuhrman grade 3~4, and lymphovascular invasion. Although the predictive accuracy was excellent, the model was developed based on only 95 cases and validated by 200 times bootstrap resample, which could cause overfitting to some extent. Another limitation of the study is the lack of the information regarding chemotherapy and radiotherapy, which also plays an important role in evaluating the prognosis in patients with CDRCC. Thus, our study was incorporated into the nomograms regarding not only the clinicopathological characteristics but also the treatment modality. Our hope is that the established nomograms could provide a more comprehensively prognostic evaluation for such rare disease.

This study has still many limitations. Firstly, the established models were based on a secondary analysis on a publicly available database. Previous studies suggested that CDRCC is difficult to be differentiated from other histologic subtypes like medullary RCC and urothelial papillary carcinoma [31, 32]. Thus, the lack of centralized pathology review may cause misclassification in the study cohort, which limits the quality of the data. Secondly, the SEER database does not provide information on patients' comorbidity such as ASA score, Eastern Cooperative Oncology Group performance status (ECOG-PS), Karnofsky score, blood parameters, and details of the adjuvant chemotherapy regimen and the completion rate, which may also be associated with patients' prognosis. Thirdly, the nomograms were only validated using bootstrap validation due to the rarity of this population. Further studies are needed to externally validate the proposed nomograms.

## 5. Conclusions

In conclusion, this study investigated a relatively large cohort of CDRCC patients using the SEER database and analyzed the prognostic factors associated with prognosis. Finally, a survival prediction nomogram was described that can make an individualized evaluation of OS and CSS in patients with CDRCC, which could help urologists to perform a better risk stratification.

## Figures and Tables

**Table 1:** The clinicopathologic characteristics of our study cohort.

Variable	All cohort (*N* = 324)
Sex	
Male	223 (68.8)
Female	101 (31.2)
Age (years), IQR	61.5 (53~72)
Year of diagnosis	
2004~2009	191 (59.0)
2010~2015	133 (41.0)
Marital status	
Unmarried	113 (34.9)
Married	199 (61.4)
Unknown	12 (3.7)
Race	
White	227 (70.1)
Black	73 (22.5)
Others	24 (7.4)
Tumor size (cm), IQR	6 (4~8.43)
Tumor side	
Left	169 (52.2)
Right	155 (47.8)
Tumor grade	
G1	10 (3.1)
G2	37 (11.4)
G3	122 (37.7)
G4	83 (25.6)
Gx	72 (22.2)
T stage	
T1	97 (29.9)
T2	18 (5.6)
T3	122 (37.7)
T4	83 (25.6)
Tx	4 (1.2)
N stage	
N0	198 (61.1)
N1	116 (35.8)
Nx	10 (3.1)
M stage	
M0	205 (63.3)
M1	115 (35.5)
Mx	4 (1.2)
AJCC stage	
I	80 (24.7)
II	14 (4.3)
III	66 (20.4)
IV	158 (48.8)
Unknown	6 (1.8)
Surgical type	
Without	44 (13.6)
NSS	20 (5.1)
RN	260 (81.3)
Radiotherapy	
Without	288 (88.9)
With	36 (11.1)
Chemotherapy	
Without	236 (72.8)
With	88 (27.2)
Follow-up time (months) IQR	17 (6~55.8)
Endpoint	
Death	249 (76.9)
Cancer-specific death	208 (64.2)

IQR: interquartile range.

**Table 2:** Univariate and multivariate Cox regression models associated with overall survival.

Variable	Univariate analysis			Multivariate analysis		
	HR	95% CI	*P*	HR	95% CI	*P*
Sex						
Male	Ref					
Female	0.903	0.689~1.183	0.460			
Age	1.012	1.003~1.022	0.011	1.016	1.006~1.026	0.002
Marital status						
Unmarried	Ref					
Married	1.126	0.862~1.472	0.383			
Race						
White	Ref					
Black	0.861	0.635~1.168	0.336			
Others	1.038	0.638~1.689	0.881			
Tumor size	1.031	1.017~1.044	<0.001	1.002	1.000~1.004	0.014
Tumor side						
Left	Ref					
Right	1.191	0.929~1.527	0.168			
Tumor grade						
G1	Ref			Ref		
G2	1.798	0.532~6.078	0.345	1.139	0.333~3.901	0.836
G3	4.580	1.449~14.479	0.010	2.090	0.645~6.771	0.219
G4	5.255	1.650~16.737	0.005	2.722	1.002~8.895	0.043
Gx	4.995	1.564~15.955	0.007	2.066	0.628~6.799	0.232
T stage						
T1	Ref			Ref		
T2	1.328	0.757~2.328	0.322	NA	NA	0.675
T3	2.308	1.711~3.113	<0.001	NA	NA	0.107
T4	3.475	2.191~5.512	<0.001	NA	NA	0.399
Tx	4.192	1.514~11.607	0.006	NA	NA	0.355
N stage						
N0	Ref			Ref		
N1	2.591	1.986~3.379	<0.001	1.645	1.202~2.252	0.002
Nx	3.894	2.027~7.480	<0.001	2.549	1.187~5.475	0.016
M stage						
M0	Ref			Ref		
M1	4.115	3.141~5.390	<0.001	3.724	2.623~5.288	<0.001
Mx	5.183	1.900~14.135	<0.001	1.141	0.350~3.724	0.827
Surgical type						
Without	Ref			Ref		
NSS	0.078	0.035~0.176	<0.001	0.224	0.094~0.535	0.001
RN	0.312	0.222~0.438	<0.001	0.515	0.339~0.781	0.002
Radiotherapy						
Without	Ref			Ref		
With	2.238	1.561~3.208	<0.001	NA	NA	0.822
Chemotherapy						
Without	Ref			Ref		
With	1.725	1.313~2.268	<0.001	0.512	0.357~0.735	0.014

**Table 3:** Univariate and multivariate Cox regression models associated with cancer-specific survival.

Variable	Univariate analysis			Multivariate analysis		
	HR	95% CI	*P*	HR	95% CI	*P*
Sex						
Male	Ref					
Female	0.808	0.597~1.092	0.165			
Age	1.003	0.993~1.014	0.514			
Marital status						
Unmarried	Ref			Ref		
Married	1.371	1.013~1.856	0.041	NA	NA	0.361
Race						
White	Ref					
Black	0.797	0.566~1.123	0.194			
Others	1.096	0.663~1.812	0.722			
Tumor size	1.033	1.020~1.047	<0.001	1.002	1.000~1.004	0.026
Tumor side						
Left	Ref					
Right	1.142	0.870~1.499	0.340			
Tumor grade						
G1	Ref			Ref		
G2	2.451	0.310~19.346	0.395	1.484	0.186~11.849	0.709
G3	11.387	1.584~81.864	0.016	4.462	0.608~32.727	0.141
G4	12.855	1.780~92.818	0.013	5.566	1.003~41.049	0.029
Gx	11.396	1.572~82.606	0.016	4.493	0.606~33.300	0.142
T stage						
T1	Ref			Ref		
T2	1.349	0.718~2.536	0.352	NA	NA	0.615
T3	2.560	1.830~3.582	<0.001	NA	NA	0.075
T4	3.764	2.280~6.214	<0.001	NA	NA	0.395
Tx	2.531	0.612~10.474	0.200	NA	NA	0.117
N stage						
N0	Ref			Ref		
N1	2.920	2.195~3.883	<0.001	1.638	1.182~2.271	0.003
Nx	2.777	1.210~6.373	0.016	1.989	0.748~5.290	0.168
M stage						
M0	Ref			Ref		
M1	4.977	3.721~6.657	<0.001	4.169	2.870~6.055	<0.001
Mx	4.912	1.545~15.613	0.007	1.551	0.396~6.074	0.529
Surgical type						
Without	Ref			Ref		
NSS	0.043	0.013~0.139	<0.001	0.160	0.047~0.547	0.004
RN	0.314	0.219~0.450	<0.001	0.553	0.357~0.858	0.008
Radiotherapy						
Without	Ref			Ref		
With	2.285	1.557~3.353	<0.001	NA	NA	0.561
Chemotherapy						
Without	Ref			Ref		
With	2.069	1.553~2.757	<0.001	0.497	0.343~0.720	<0.001

## Data Availability

All the data in the current study are publicly available in the Surveillance, Epidemiology, and End Results database (https://seer.cancer.gov/).

## References

[1] Siegel R. L., Miller K. D., Fuchs H. E., Jemal A. (2021). Cancer statistics, 2021. *CA: a Cancer Journal for Clinicians*.

[2] Patard J. J., Leray E., Rioux-Leclercq N. (2005). Prognostic value of histologic subtypes in renal cell carcinoma: a multicenter experience. *Journal of Clinical Oncology*.

[3] Leibovich B. C., Lohse C. M., Cheville J. C. (2018). Predicting oncologic outcomes in renal cell carcinoma after surgery. *European Urology*.

[4] Zisman A., Pantuck A. J., Dorey F. (2001). Improved prognostication of renal cell carcinoma using an integrated staging system. *Journal of Clinical Oncology*.

[5] Frank I., Blute M. L., Cheville J. C., Lohse C. M., Weaver A. L., Zincke H. (2002). An outcome prediction model for patients with clear cell renal cell carcinoma treated with radical nephrectomy based on tumor stage, size, grade and necrosis: the SSIGN score. *The Journal of Urology*.

[6] Leibovich B. C., Blute M. L., Cheville J. C. (2003). Prediction of progression after radical nephrectomy for patients with clear cell renal cell carcinoma: a stratification tool for prospective clinical trials. *Cancer*.

[7] Amin M. B., Edge S. B., Greene F. L. (2017). *The 8th Edition of the AJCC Cancer Staging Manual*.

[8] Wright J. L., Risk M. C., Hotaling J., Lin D. W. (2009). Effect of collecting duct histology on renal cell cancer outcome. *The Journal of Urology*.

[9] Sui W., Matulay J. T., Robins D. J. (2017). Collecting duct carcinoma of the kidney: disease characteristics and treatment outcomes from the National Cancer Database. *Urologic Oncology*.

[10] May M., Ficarra V., Shariat S. F. (2013). Impact of clinical and histopathological parameters on disease specific survival in patients with collecting duct renal cell carcinoma: development of a disease specific risk model. *The Journal of Urology*.

[11] Tokuda N., Naito S., Matsuzaki O. (2006). Collecting duct (Bellini duct) renal cell carcinoma: a nationwide survey in Japan. *The Journal of Urology*.

[12] Klang E., Lawson P., Yonat H., Michal Amitai M. (2017). Metastatic collecting (Bellini) duct carcinoma presented in a young patient: a case report and review of the literature. *The Israel Medical Association Journal*.

[13] Hasan A., Abozied H., Youssef A., Fayad S., Ismail A. (2020). A rare case of collecting duct carcinoma with first presentation of respiratory symptoms. *Urology case reports*.

[14] Oueslati A., Saadi A., Chakroun M. (2020). Bellini duct carcinoma revealed by cutaneous metastasis: a case report. *International Journal of Surgery Case Reports*.

[15] Deuker M., Stolzenbach F., Rosiello G. (2020). Renal cell carcinoma: comparison between variant histology and clear cell carcinoma across all stages and treatment modalities. *The Journal of Urology*.

[16] Karakiewicz P. I., Trinh Q. D., Rioux-Leclercq N. (2007). Collecting duct renal cell carcinoma: a matched analysis of 41 cases. *European Urology*.

[17] Steyerberg E. W., Vergouwe Y. (2014). Towards better clinical prediction models: seven steps for development and an ABCD for validation. *European Heart Journal*.

[18] Abern M. R., Tsivian M., Polascik T. J., Coogan C. L. (2012). Characteristics and outcomes of tumors arising from the distal nephron. *Urology*.

[19] Oudard S., Banu E., Vieillefond A. (2007). Prospective multicenter phase II study of gemcitabine plus platinum salt for metastatic collecting duct carcinoma: results of a GETUG (Groupe d'Etudes des Tumeurs Uro-Génitales) study. *The Journal of Urology*.

[20] Pécuchet N., Bigot F., Gachet J. (2013). Triple combination of bevacizumab, gemcitabine and platinum salt in metastatic collecting duct carcinoma. *Annals of Oncology*.

[21] Sheng X., Cao D., Yuan J. (2018). Sorafenib in combination with gemcitabine plus cisplatin chemotherapy in metastatic renal collecting duct carcinoma: a prospective, multicentre, single-arm, phase 2 study. *European Journal of Cancer*.

[22] Fleming S., Lewi H. J. (1986). Collecting duct carcinoma of the kidney. *Histopathology*.

[23] Mennitto A., Verzoni E., Peverelli G., Alessi A., Procopio G. (2018). Management of metastatic collecting duct carcinoma: an encouraging result in a patient treated with cabozantinib. *Clinical Genitourinary Cancer*.

[24] Yasuoka S., Hamasaki T., Kuribayashi E. (2018). Nivolumab therapy for metastatic collecting duct carcinoma after nephrectomy: a case report. *Medicine*.

[25] Watanabe K., Sugiyama T., Otsuka A., Miyake H. (2020). Complete response to combination therapy with nivolumab and ipilimumab for metastatic collecting duct carcinoma of the kidney. *International Cancer Conference Journal*.

[26] Zeng Y., Zhang W., Li Z. (2020). Personalized neoantigen-based immunotherapy for advanced collecting duct carcinoma: case report. *Journal for Immunotherapy of Cancer*.

[27] Bronchud M. H., Castillo S., Escriva de Romaní S. (2012). HER2 blockade in metastatic collecting duct carcinoma (CDC) of the kidney: a case report. *Onkologie*.

[28] Pagani F., Colecchia M., Sepe P. (2019). Collecting ducts carcinoma: An orphan disease. Literature overview and future perspectives. *Cancer treatment reviews*.

[29] NCT02363751.

[30] NCT03354884.

[31] el Khoury J., Abdessater M., Halabi R. (2020). Bellini duct carcinoma misdiagnosed with urothelial papillary carcinoma. *Case Reports in Oncological Medicine*.

[32] Ohe C., Smith S. C., Sirohi D. (2018). Reappraisal of morphologic differences between renal medullary carcinoma, collecting duct carcinoma, and fumarate hydratase-deficient renal cell carcinoma. *The American Journal of Surgical Pathology*.

